# Supplementary material for: The genome sequence of long-finned pilot whale, Globicephala melas (Traill, 1809)
Source: Wellcome Open Res. 2025 Apr 8;10:180. [Version 1] doi: 10.12688/wellcomeopenres.23919.1 (PMC12009481; doi:10.12688/wellcomeopenres.23919.1)
Supplement: Peer review history

# Reviewer report: Reviewer response for version 1

**DOI:** 10.21956/wellcomeopenres.26388.r122148

**Published:** 2025-04-19

**Authors:** Janke Axel

**Affiliations:** 1 Ecological Genomics, BiK-F/Goethe University/Senckenberg, Frankfurt, Germany

**Peer Review Comments -  MAJOR REVISION**

 This manuscript presents a genome report which, while lacking a specific biological research question, provides a technically sound account of genome sequencing, assembly, and annotation. The work is professionally conducted, and the methodology is mostly described with sufficient clarity.

 However, several important aspects are either missing or require further elaboration to ensure the study's utility for the broader community, particularly for those working on similar non-model or degraded samples.

**1. DNA and RNA Quality Assessment:**

 There is no information provided on the quality or integrity of the extracted DNA. Given that the sample appear to originate from a naturally deceased animal, with an unknown post-mortem interval, this is of critical importance. Please include the Femto-Pulse report or equivalent data showing DNA fragment size distribution. This information is vital to assess the potential limitations of the assembly and to inform future efforts involving similarly compromised material. The same applies to the RNA quality: please provide fragment length distribution and RIN-like metrics for evaluation of transcript integrity. These details are partcularly valuable when fresh or high-quality material is not available.

**2. Blob Plot Interpretation:**

 The Blob plot reveals a number of non-target sequences, likely corresponding to bacterial or fungal contamination. While such findings are not unexpected given the possible post-mortem degradation, the manuscript should briefly elaborate on the origin, taxonomy, and abundance of these non-mammalian sequences. It would be helpful to state whether these were filtered prior to assembly or if any were incorporated into scaffolds. This information may help guide decontamination strategies in similar projects.

**3. Sample Context and Decay Estimate:**

 To support the interpretation of the data, please provide an estimate—however approximate—of how long the individual had been deceased prior to sampling. Environmental context (e.g., temperature, exposure, humidity) should also be described to the best of the authors’ knowledge. This would help frame the state of preservation and assist others in evaluating feasibility for related work using non-ideal specimens.

**4. Chromosome-Specific Concerns (Chromosome 6):**

 The heatmap corresponding to the sixth(?) chromosome suggests structural anomalies or misassemblies. Please describe whether any steps were taken investigate or correct these issues (e.g., manual curation, re-scaffolding, use of alternative data types). If no specific action was taken, please clarify why. Similarly, assemblies not in the heatmap should be briefly addressed: were they omitted due to quality, completeness, or other reasons? Even a short rationale would be appreciated.

**5. Background Section:**

 The current background is overly detailed relative to the scope of the manuscript. As no biological analyses or questions are pursued, it would be preferable to reduce this section to the essential taxonomic and minimal biological context required to understand the importance of the project.

**Conclusion:**

 While the manuscript lacks any biological hypothesis (read: it is boring), it still holds potential value for the community, especially as a reference for genome sequencing from compromised material. By addressing the issues above, the manuscript will be significantly improved and maybe more useful except for another genome . Still, I am looking forward to seeing it indexed.

Are sufficient details of methods and materials provided to allow replication by others?

Partly

Is the rationale for creating the dataset(s) clearly described?

Partly

Are the datasets clearly presented in a useable and accessible format?

Partly

Are the protocols appropriate and is the work technically sound?

Partly

Reviewer Expertise:

Translational Biodiversity Genomics

I confirm that I have read this submission and believe that I have an appropriate level of expertise to confirm that it is of an acceptable scientific standard, however I have significant reservations, as outlined above.

# Reviewer report: Reviewer response for version 1

**DOI:** 10.21956/wellcomeopenres.26388.r122154

**Published:** 2025-04-19

**Authors:** Takushi Kishida

**Affiliations:** 1 Nihon University, Kanagawa, Japan

This study provides a high-quality chromosomal-level long-finnedpilot whale haploid genome assembly. I have no complaints about the quality of this genome data.

 I hope, if possible, the authors deposit the specimen used for genome sequencing in an appropriate museum so that a vouchered specimen associated with this genome data is available.

Are sufficient details of methods and materials provided to allow replication by others?

Yes

Is the rationale for creating the dataset(s) clearly described?

Yes

Are the datasets clearly presented in a useable and accessible format?

Yes

Are the protocols appropriate and is the work technically sound?

Yes

Reviewer Expertise:

evolutioary genomics, population genetics

I confirm that I have read this submission and believe that I have an appropriate level of expertise to confirm that it is of an acceptable scientific standard.

# Reviewer report: Reviewer response for version 1

**DOI:** 10.21956/wellcomeopenres.26388.r122151

**Published:** 2025-04-18

**Authors:** Isabella M Reeves

**Affiliations:** 1 Flinders University,, Bedford Park, Australia

This manuscript is an exciting contribution to our field, providing a high-quality genome assembly of the long-finned pilot whale for future research to use. The introduction, which covers the natural history of pilot whales, was particularly engaging.

 One suggestion I would like to offer is the inclusion of more detailed information regarding the software and tools used during the genome assembly process. Specifically, it would be beneficial to provide the versions of the software employed, as well as additional details on the parameters/thresholds used throughout the assembly.

Are sufficient details of methods and materials provided to allow replication by others?

Partly

Is the rationale for creating the dataset(s) clearly described?

Yes

Are the datasets clearly presented in a useable and accessible format?

Yes

Are the protocols appropriate and is the work technically sound?

Yes

Reviewer Expertise:

Evolution, genomics, marine mammals, ecology

I confirm that I have read this submission and believe that I have an appropriate level of expertise to confirm that it is of an acceptable scientific standard.
